# Why do parents refuse childhood vaccination? Reasons reported in Finland

**DOI:** 10.1177/14034948211004323

**Published:** 2021-04-12

**Authors:** Johanna Nurmi, Bronwyn Harman

**Affiliations:** 1 University of Turku, Turku, Finland; 2 Edith Cowan University, Joondalup, Australia

**Keywords:** Vaccination, immunization, vaccine hesitancy, vaccination refusal, parents, health perceptions, health practices

## Abstract

**Aims:**

This article examines the reasons for partial and complete refusal of childhood vaccination as reported by parents in Finland. It analyzes perceptions and experiences central in vaccination decisions.

**Methods:**

The analysis is based on 38 in-depth interviews with Finnish parents who have refused all or several vaccines for their children. The interviews were analyzed using qualitative content analysis.

**Results:**

Three categories of reasons were identified in the analysis: 1) risks and effects of vaccination – concern about and/or experiences of possible side-effects was the most important reason for avoiding vaccines; 2) distrust – participants did not trust vaccination recommendations made by health officials and medical professionals due to perceived bias in medical research, ties between health officials and the pharmaceutical industry, and personal experiences of (suspected) adverse effects and the way these concerns were received in healthcare institutions; 3) health perceptions and practices – parents supported their vaccination choices with complementary and alternative medicine treatments and alternative health understandings. Many stated that contracting vaccine-preventable illnesses would provide longer lasting and more ‘natural’ immunity than vaccination, and possibly other health benefits.

**Conclusions:**

**A loss of trust in medical and public health actors was central to the process in which parents came to question, contest, and eventually refuse childhood vaccination. The adverse effects of the Pandemrix vaccine in 2009–2010 have been important in leading to distrust and contestation. Distrust may relate to personal experiences of (suspected) adverse effects or to broader concerns over the neutrality of health authorities and the trustworthiness of medical research.**

## Background

The growing number of parents who question vaccination recommendations, along with increasingly critical attitudes toward vaccination, have caused concern around the globe [1–3]. Social and public health research has grouped the diverse attitudes that question or critique immunization under the term ‘vaccine hesitancy’ (e.g. Larson et al. [1]). Vaccine refusal is part of the phenomenon of vaccine hesitancy, which consists of individuals questioning, delaying, and refusing some or all vaccines, or accepting vaccines but being unsure of their decision to do so [1]. Research has identified factors such as fear of adverse effects, negative experiences related to vaccination, and lack of trust in the efficacy of vaccines as possible reasons for parental contestation of vaccination [4–7]. Several analyses have also linked vaccine hesitancy and refusal to gendered neoliberal parenting discourses that expect individuals to be responsible for their own wellbeing, make healthy choices and manage their children’s health [6,8,9].

Because parents’ reasons for questioning vaccination are complex and context-specific, research needs to explore how vaccine hesitancy and refusal emerge in particular times and places [10]. Until now, research has concentrated on North America and Europe (excluding the Nordic countries) [1]. Although some studies on parental attitudes toward childhood vaccines in general [11] or individual vaccines such as the rotavirus, HPV or MMR vaccines [12–16] have been conducted in the Nordic Countries, the overall reasons that parents report for refusing childhood vaccines have not been mapped.

This article examines the reasons that parents state for refusing several or all recommended childhood vaccines in Finland. The uptake of the basic childhood vaccines (MMR, DtaP-IPV-Hib, Rotavirus, and Pneumococcal conjugate) in the country is high (92.5–98.4%) [17]. Only 1% of Finnish children under the age of three have not received any of the basic vaccines [18]. However, the vaccination uptake for the HPV vaccine (70% of girls) and seasonal influenza (43% of children aged 6–35 months) is remarkably lower [19,20].

Overwhelmingly positive attitudes toward vaccination are reported in the Nordic countries [21,22], although a survey in 2018 noted that confidence in the safety of vaccines had decreased in Finland and Sweden compared to 2015 [22]. According to a recent survey, Finns place a great amount of trust in state institutions, scientific institutions, the judicial system, and science in general; 95% of respondents completely or mostly agreed that the vaccines used in Finland are effective and safe. Similarly, 89% reported trust in the vaccine information provided by experts and authorities [23].

However, as many as 32% of respondents in the same survey completely or mostly agreed that the side-effects of vaccines are not discussed enough [23]. Moreover, 13% agreed that vaccines are given to children because it is profitable for the pharmaceutical industry. A certain level of distrust in vaccination thus exists in Finland, and some citizens worry about their possible harmfulness. Still, the overall high levels of trust and vaccination uptake make Finland an interesting case for looking into why some parents refuse immunization in a cultural atmosphere of trust and appreciation toward vaccination.

## Aim

This article examines the reasons for partial and complete refusal of childhood vaccination as reported by parents in Finland. It aims to provide an overview of the main perceptions and experiences which are central in the immunization decisions of parents who opt out of some or all childhood vaccines.

## Methods

### Participants and setting

The analysis is based on 38 in-depth interviews with Finnish parents of partially vaccinated or non-vaccinated children. Participants lived in southern, western, and central Finland. All but three of the participants were women. Participants’ children were between the ages of two months and 30 years, but most of the children were minors. Even though some of the participants had adult children, all but one participant also had younger children or adult children (18–20 years old) living at home. The participants had a total of 106 children, of which 45 were non-vaccinated, 37 were partially vaccinated, and 24 were fully vaccinated until at least the age of six. Some continued to give their children certain recommended vaccines. Six participants had never vaccinated any of their children. Some considered their vaccination decisions to be fairly permanent, while others stated that they might reconsider vaccination later.

### Data collection

Parents who refuse childhood vaccination are often hesitant to participate in studies due to their marginalized position in a cultural context of high trust in vaccination and high rates of compliance with vaccination recommendations. Purposeful sampling [24] was thus used to ensure an adequate number of participants who had opted out of some or all recommended vaccines for at least one of their children.

Participants were first recruited with an invitation posted to an open Finnish vaccine-skeptic Facebook group. Those who participated were asked to refer other participants who might not have seen the Facebook invitation or who might hesitate. This method reached people who were connected (on Facebook or through personal connections) with other parents of partially or non-vaccinated children. The interviews were conducted by the first author in 2016–2019. Data collection ended after saturation was reached and several parents of partially and non-vaccinated children had participated, as well as parents of children with diagnosed, suspected, and no side-effects.

Most interviews were conducted at the participants’ homes, although three were conducted at cafés and two took place over the phone at the participants’ request. The interviews covered three major themes: the experiences and reasons that had led participants not to vaccinate their child(ren), their health perceptions and practices, and their encounters with healthcare professionals. Background information (year of birth, education, profession, age and profession of possible spouse, ages of children) was also collected. Interviews were recorded and transcribed verbatim. The study followed the guidelines of the Finnish National Board on Research Integrity. Participants provided written consent for the interviews, and the names used in this article are pseudonyms. According to the guidelines of the Ethics Committee for Human Sciences of the University of Turku, an ethical review of the study was not required.

### Analysis

The interviews were analyzed using qualitative content analysis. The creation of coding categories was informed but not determined by the concepts derived from the existing social research on vaccination presented in the previous section (i.e. trust, distrust, individual responsibility). The reasons reported by the parents for partial or non-vaccination were first coded into five different categories: 1) side-effects, 2) risks and benefits, 3) distrust, 4) health perceptions and practices, and 5) broad-scale consequences. Later, some of the codes were merged because of similarities, resulting in three categories of reasons that will be presented in the following section in order of importance (most mentions): 1) risks and effects of vaccination, 2) distrust, and 3) health perceptions and practices. This paper thus provides a general overview of reasons for vaccine refusal stated by parents in Finland.

## Findings

### Risks and effects of vaccination

Concern about the possible side-effects of vaccination was the most important reason for avoiding vaccines; it was mentioned the most, and many explicitly cited the risk of adverse effects as their number one reason for not vaccinating. Participants typically referred to serious, rare, and contested symptoms rather than the common mild reactions that occur after vaccination.

Most participants talked about their own experiences with side-effects which afflicted themselves or their children. Six participants had children who were diagnosed by medical doctors with serious adverse effects or an illness connected to vaccination. One had lost their child due to an illness induced by vaccination. However, most of the problems experienced by the participants or their children (such as allergies, autism, asthma, dysphasia, and digestive problems) were not confirmed as vaccination-related by medical professionals, even if participants strongly suspected a link. Still, the experience of a suspected side-effect was usually so strong that it became central in parents’ reasoning, often overriding healthcare workers’ assurances that their child’s condition was not caused by vaccines. This was the case with Mia, whose one-year-old son had a large vocabulary for his age but stopped talking soon after vaccination. Two years later, he was still not talking at the same level as before. Mia strongly suspected a link between vaccination and his loss of speech, but this was not validated by their nurse at the public child health clinic:

They think it can’t be because of this vaccine, but that it was caused by him learning to walk. But he started walking at one year and one month, and all the words had already disappeared by that time. Then they said it was because his little sister was born. But from the time he started walking it was more than six months until his sister was born. (Mia)

Mia then discontinued vaccination. It was common for participants to stop vaccinating their children after the occurrence of diagnosed or suspected side-effects.

Several participants had witnessed side-effects experienced by family or friends or had heard of other people’s experiences from acquaintances or through social media. However, the suspected link between autism and the MMR vaccine, which is a common concern of parents who refuse vaccines in English-speaking countries, was mentioned by only a few participants. Most were careful not to claim that the MMR vaccine caused autism. Instead, for many participants, it was the influenza vaccine Pandemrix and the related narcolepsy cases that made them start questioning vaccination and the trustworthiness of health authorities.

In 2009–2010, half the population of Finland was vaccinated against H1N1 influenza. Soon after, there was a sudden increase in children diagnosed with narcolepsy, and, later, the link between the Pandemrix vaccine and an increased risk of narcolepsy was recognized [25]. Several participants described the confirmed link between narcolepsy and vaccination as the ‘wake-up call’ that initially made them question vaccination. For others, it was proof that they were on the right track avoiding vaccination.

Many participants’ understandings of the risks and benefits of vaccination differed drastically from the official public health discourse. They said that vaccines were ineffective in preventing diseases – an assertion that was often based on themselves or a family member contracting a vaccine-preventable disease (VPD) despite being immunized. Rather than basing their decisions on the recommendations of health officials, parents were drawing from personal experiences, national-level or local events related to vaccination, and information gathered from various sources.

### Distrust

The second category of reasons was related to distrust toward health authorities, medical research on vaccination, or healthcare providers. Often, the lack of trust stemmed from the role of the market economy and financial interests in healthcare and vaccination, and this was the perspective from which participants interpreted information they gathered from scientific articles, media, health officials’ materials, vaccine-critical online material, social media discussions, and other sources.

While many participants said they read scientific articles on vaccine safety, they were often distrustful toward medical studies on vaccines. Overwhelmingly, they criticized the fact that pharmaceutical companies fund and conduct studies on vaccine safety and efficacy, asserting that these studies are not impartial because of financial interest: ‘What they study and what the hypotheses are, it’s tied to money. That’s why I feel that reading these studies doesn’t give me much [information]’ (Jenny). Participants cited examples of pharmaceutical companies only publishing results that ‘look good for the product’, potentially hiding problems in vaccine safety: ‘It worries me. These are big corporations, but they work in secrecy’ (Hanna).

Participants also criticized study designs comparing new vaccines with older ones, stating that only studies using double-blinded placebo, where the placebo would not contain any adjuvants, can provide accurate information about vaccine safety. Many called for longitudinal studies comparing the prevalence of suspected long-term consequences of vaccines (such as allergies) in vaccinated and non-vaccinated populations. Like Tom, a father of five, many also criticized health officials for relying on research performed by pharmaceutical companies: ‘I’m most disappointed in the health officials because they don’t do their own research, they just look at studies that are usually always done by the manufacturer of the vaccine. That makes it questionable for me’ (Tom). This quote illustrates that participants were not denying science per se but calling for more independent research. Thus, participants felt that it was almost impossible to find independent and impartial information about vaccines, and felt it was better to avoid vaccination.

Most participants criticized ties between the pharmaceutical industry and health officials. Many pointed out that the National Institute for Health and Welfare (THL), which steers the national vaccination program, has received research funding from GlaxoSmithKline [26]. They also pointed out instances of ‘revolving doors’ in which individuals who previously worked in pharmaceutical companies were hired as public health officials. The fact that Finland purchased the Pandemrix H1N1 vaccine from GlaxoSmithKline in 2009 was used as an example of how industry ties may affect public decision-making. For instance, Leo, whose child was diagnosed with narcolepsy after being administered the Pandemrix vaccine, felt that industry collaboration was a relevant factor in the ‘narcolepsy scandal’. Subsequently, participants said they were unable to trust the vaccination recommendations of health officials.

Participants had also experienced distrust in health officials during the campaign for the Pandemrix vaccine in the winter of 2009–2010. For instance, Jessica, who had never vaccinated her two young children, stated that ‘my spouse practically doesn’t trust any [information] that comes from the official actors, and my trust toward THL [the National Institute for Health and Welfare] has been diminished quite a lot by this issue of the swine flu’. Some participants accused health officials of fear mongering and pressuring people to vaccinate. The health officials had communicated that the vaccine was safe and H1N1 influenza was dangerous. When it turned out that the vaccine increased the risk of narcolepsy and the H1N1 influenza was less lethal than originally feared, these participants felt betrayed.

Participants were not only distrustful of pharmaceutical companies and health authorities, but many had trouble trusting healthcare institutions and even individual healthcare professionals. While many participants who talked about distrust had not suspected side-effects in their own children, there were some, such as Mia (whose child had stopped talking after vaccination), whose distrust stemmed from their experiences of possible vaccine-related side-effects being dismissed without investigation by doctors or nurses. Moreover, those who had experienced diagnosed, severe adverse effects strongly criticized the state for its lack of adequate compensation and support.

### Health perceptions and practices

Participants also presented health-related perceptions and practices as reasons for vaccine refusal. They often stated views and attitudes alternative to the mainstream understanding of health and illness; for instance, they talked about VPDs serving a purpose in strengthening the immune system. Vaccination, on the contrary, was not seen as natural at all – especially combination vaccines: ‘It’s not natural, so it can’t be good for us’ (Lea), was an argument repeated by many.

Many participants hoped that their children would get illnesses such as chickenpox or measles during childhood when the symptoms would allegedly be milder; contracting the illness would also provide longer lasting, more ‘natural’ immunity than vaccination, and could possibly provide other health benefits:

There’s indications that having certain illnesses will protect you from others. I found a study that said that children who’ve had the rotavirus had significantly lower rates of severe respiratory illnesses and pneumonia. Then I’ve read about measles – that it has (. . .) a protective effect against certain types of cancer, same with mumps (. . .) It may be nature’s way of strengthening your immunity so that you’ll live longer and be healthier. (Irene)

These findings also resonate with a study on Swedish anthroposophic parents who perceived measles as strengthening [12].

Participants often named complementary and alternative medicine (CAM) as an important part of their own set of practices related to illness prevention. Some had been told by CAM practitioners that vaccination was unnecessary or harmful. Most participants used CAM treatments for their children, although all consulted medical professionals when necessary. Many stated that CAM treatments – especially homeopathy – provided them with tools to both prevent and treat illnesses, including VPDs. They also used other health practices such as nutrition, long-term breastfeeding, and the building of healthy gut flora as ways to support the immune system.

## Discussion

The most important reason stated by the participants for vaccine refusal was the potential harm caused by vaccines. Secondly, issues of distrust also gained considerable importance in participants’ accounts. Thirdly, parents supported their vaccination choices with CAM treatments and alternative health perceptions.

For many participants, the H1N1 influenza pandemic and the adverse effects of the related vaccine were important in creating distrust and contestation of vaccination. Specific concerns and contestations in fact emerge in connection to geopolitical and historical contexts of (mis)trust between the state and citizens. This has been shown in recent analyses of failed vaccination campaigns in countries with lower institutional trust, such as Romania and Ukraine [27,28]. However, in the Nordic countries, general trust in state officials and institutions is high and this trust extends to public health officials and vaccination programs [21–23]. Moreover, the Nordic countries, Finland included, do not have strong ‘anti-vaccination movements’. This is in contrast to other counties such as the US, the UK, or Australia where such movements have been influencing public discussion and public opinion for the past several decades [29].

In Finland, despite high levels of institutional trust, a unique context of distrusts was created by revelations about the side-effects of the Pandemrix vaccine and the actions and statements of state and public health actors in response to those revelations. This distrust was reflected in the decreased uptake of influenza vaccines in the years after the Pandemrix-related narcolepsy cases, and can perhaps still be seen in the lower uptake levels of children’s influenza vaccines and the HPV vaccine, which is perceived as a ‘new’ vaccine [19,20]. As we have shown, the distrust created by the Pandemrix-related narcolepsy was also still visible in the accounts of the participants of this study. Another example can be found in Denmark, where a decrease in the HPV vaccination rate has been connected to public concern and media coverage about the vaccine’s possible side-effects [30].

However, trust in vaccination should not be understood as merely a means to increase vaccination uptake, but as ‘the result of good, ethically justified, public health activities’ [31]. Respectful dialogue – both in public discussion and in clinical encounters – with groups contesting childhood vaccination, as well as transparency and limited industry collaboration by the main public health actors responsible for the vaccination program, could encourage trust within critical and hesitant groups.

While concern has arisen about the persuasive narratives of the negative vaccination experiences diffused by the anti-vaccine movement(s) [32], parents in this study stressed personal experiences of (suspected) adverse effects and general feelings of distrust toward the actors involved in vaccine development and policies as more persuasive. In fact, some globally circulating arguments against vaccination, such as the suggested MMR–autism connection, may have become unappealing in Finland, namely because of their connection with anti-vaccine movements. The majority of the population maintains trust in vaccination and public health officials, and public discussion about vaccine refusal in Finland has included disparaging remarks which characterize non-vaccinating parents as lacking in intelligence, not understanding science, and gullible to conspiracy theories. This may have led participants to present themselves as individuals who understand the principles of scientific research and offer concrete criticism rather than vague claims about ‘big pharma’ and corruption.

This study has some limitations related to data collection. The study only reached participants who were connected to a loose network of individuals critical of vaccination. There may be other Finnish parents who have decided not to vaccinate without support from such networks. Findings are thus not generalizable to all parents in Finland who have refused some or all of their children’s vaccinations. Moreover, almost all participants were women and the perspective of fathers is thus not equally represented in the interview materials. However, most participants asserted that they had made their vaccination choices together with their partner, or that their partner agreed with the decision of (partial) non-vaccination. Another limitation is that within the scope and the analytic framework of this article, it can offer only a general overview of the reasons for vaccine refusal that the participants highlighted as the most important. However, it cannot provide a very detailed insight into the many complexities of the phenomenon and processes of vaccine refusal in the Nordic context, which remains to be addressed in future research.

## Conclusion

A loss of trust in the medical and public health actors responsible for steering the national vaccination program may be central to the process in which some parents come to question and eventually refuse childhood vaccination. Distrust may relate to personal experiences of (suspected) adverse effects and the way these suspicions are received in healthcare institutions, or to broader concerns over the neutrality of health authorities and the trustworthiness of medical research, or both.

While vaccine refusal concerns a small minority of parents in the Nordic countries, the maintaining and (re)building of trust between lay groups and health officials or healthcare institutions remains a challenge. Past experiences with the H1N1 pandemic vaccination campaigns and the related side-effects remain in the collective memory in the Nordic countries (e.g. Börjesson and Enander [33]). Thus, hesitant and critical attitudes can increase in the wider population in other situations related to infectious diseases, such as the vaccination campaign against COVID-19.
